# Molar Bud-to-Cap Transition Is Proliferation Independent

**DOI:** 10.1177/0022034519869307

**Published:** 2019-08-08

**Authors:** S. Yamada, R. Lav, J. Li, A.S. Tucker, J.B.A. Green

**Affiliations:** 1 Centre for Craniofacial Biology and Regeneration, King’s College London, London, UK

**Keywords:** developmental biology, cell biology, morphogenesis, morphometrics, tooth development, basal constriction

## Abstract

Tooth germs undergo a series of dynamic morphologic changes through bud, cap, and bell stages, in which odontogenic epithelium continuously extends into the underlying mesenchyme. During the transition from the bud stage to the cap stage, the base of the bud flattens and then bends into a cap shape whose edges are referred to as “cervical loops.” Although genetic mechanisms for cap formation have been well described, little is understood about the morphogenetic mechanisms. Computer modeling and cell trajectory tracking have suggested that the epithelial bending is driven purely by differential cell proliferation and adhesion in different parts of the tooth germ. Here, we show that, unexpectedly, inhibition of cell proliferation did not prevent bud-to-cap morphogenesis. We quantified cell shapes and actin and myosin distributions in different parts of the tooth epithelium at the critical stages and found that these are consistent with basal relaxation in the forming cervical loops and basal constriction around enamel knot at the center of the cap. Inhibition of focal adhesion kinase, which is required for basal constriction in other systems, arrested the molar explant morphogenesis at the bud stage. Together, these results show that the bud-to-cap transition is largely proliferation independent, and we propose that it is driven by classic actomyosin-driven cell shape–dependent mechanisms. We discuss how these results can be reconciled with the previous models and data.

## Introduction

Tooth formation is a well-established model for epithelial-mesenchymal signaling interactions and for ectodermal organ formation and organogenesis in general (Pispa and Thesleff 2003; Thesleff 2003). However, until recently, the morphogenetic mechanisms of tooth development and those of other ectodermal organs were poorly understood and generally described as merely “down growth” of the epithelium into the underlying mesenchyme (Pispa and Thesleff 2003; Mikkola and Millar 2006; Ten Cate et al. 2012), suggesting largely proliferation-driven mechanisms. Recent work has shown that tooth morphogenesis is more complex. The first step—the transition from epithelium (“lamina stage”) to an invaginated tooth bud—occurs through a combination of local stratification (by vertical cell division) and local contraction (by cell intercalation) of a canopy of suprabasal cells anchored to the basal lamina by flanking “shoulder” cells (Li et al. 2016; Panousopoulou and Green 2016). The contractile canopy ultimately forms the neck of the tooth bud, and this mechanism accounts for the morphogenesis from placode all the way to the late bud stage (Li et al. 2016; Panousopoulou and Green 2016).

**Figure 1.**
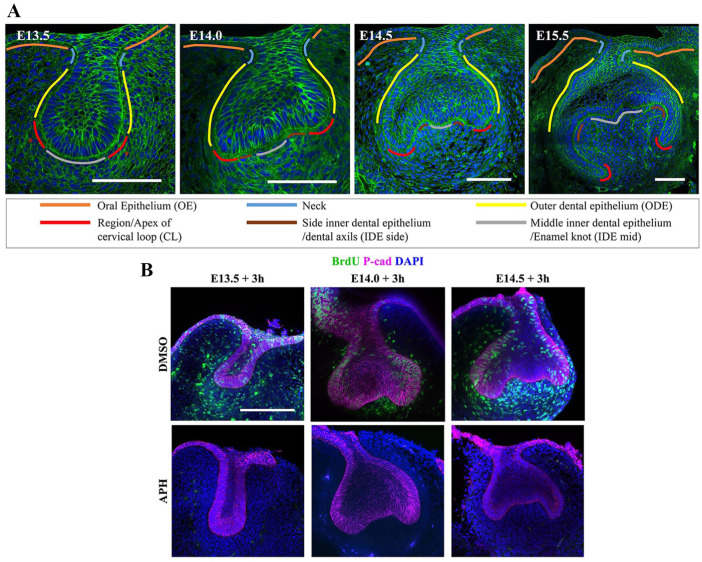
Molar tooth germs show dynamic shape change from bud to cap to bell stage. (**A**) Confocal images of frontally sliced tooth germs at the indicated stages were stained with phalloidin (green) for F-actin and DAPI (4′,6-diamidino-2-phenylindole; blue) for nuclei. Scale bar = 100 μm. (**B**) Confocal images of tooth germ explants made at the stages shown were treated with aphidicolin (APH) or vehicle as indicated, incubated for 3 h, and labeled for a further 2 h with BrdU (green). Counterstains are DAPI (nuclei, blue) and P-cadherin (magenta). Absence of green label with aphidicolin treatment shows complete inhibition of proliferation, while cells in the control group actively proliferated at 3 h of treatment. Scale bar = 100 μm for all panels.

The next step of tooth germ morphogenesis is the transition from the bud stage to the cap stage. The base of the bud flattens, forming epithelial bends known as cervical loops on either side (Fig. 1). Although known as loops because of their appearance in section, the lobes/loops are proximodistally extended ridges in molars and an annular rim in incisors (Peterkova et al. 1996; Kieffer et al. 1999). The cervical loops become gradually deeper and curve down to make the eponymous cap shape (Fig. 1) and then toward one another to make a (cow) bell shape that gives its name to the next stage. Between the cervical loops lies the inner dental epithelium (IDE), the middle of which becomes the primary enamel knot. Enamel knots are signaling centers known for having low or no cell proliferation and for being the sites of future tooth cusp formation (Jernvall et al. 1994). The correlation between low proliferation in the future cusps (epithelial peaks) and higher proliferation in the valleys—the cervical loops and intercusp regions—has given rise to models in which differential proliferation was responsible for the epithelial morphogenesis (e.g., Salazar-Ciudad and Jernvall 2002, 2010).

During the bud-to-cap transition, the surrounding mesenchyme condenses, forming a capsule. This led to ideas that epithelial proliferation within the constraining mesenchymal capsule drives the epithelium to buckle to form the cervical loop and cusps (Takigawa-Imamura et al. 2015; Morita et al. 2016). Support for this idea came from experimental removal of the mesenchyme from tooth explants at the late bud stage, which resulted in the cervical loops springing outward, showing that the mesenchyme indeed constrains the epithelial shape (Morita et al. 2016). Sophisticated live explant imaging and computer modeling showed that differential proliferation and adhesion among the mesenchymal, basal, and suprabasal epithelial cells, with constraint from less proliferative mesenchyme, could account for the bud-to-cap morphogenesis (Marin-Riera et al. 2018).

Although epithelial buckling due to proliferation within a constraining structure is known in other contexts (e.g., intestinal villus formation; Shyer et al. 2013), most known epithelial bending mechanisms rely on autonomous cell shape change, especially actin-myosin-dependent apical or basal constriction (Pearl et al. 2017). Here, we show that, contrary to prevailing tooth models, inhibition of cell proliferation does not significantly inhibit bud-to-cap morphogenesis. We quantify cell shape in the tooth epithelium at the bud stage to the cap stage and observe basal expansion in the cervical loops and basal contraction in the juxtaknot IDE. Actin and myosin staining suggests basal relaxation and active basal constriction for these, respectively. Finally, we show that inhibition of focal adhesion kinase (FAK), implicated in other instances of active basal constriction, completely inhibits bud-to-cap morphogenesis. We propose that the bud-to-cap transition is largely or entirely proliferation independent but that prior models and data are consistent with proliferation being the driver of the subsequent cap-to-bell transition.

## Materials and Methods

### Animals

Animals were handled under UK Home Office licensing and King’s College London Ethics Committee approval. Pregnant wild-type CD1 mice and mT/mG (Gt(ROSA)26Sor^tm4(ACTB-tdTomato,-EGFP)Luo^) mice (Jackson Laboratories 007576) were euthanized by cervical dislocation.

### Ex Vivo Explant Culture and Drug Treatment

Frontal slices containing tooth germs of E13.5, E14.0, and E14.5 mandibular molars were obtained as described (Alfaqeeh and Tucker 2013). Briefly, mandibles were manually dissected from the heads in Advanced Dulbecco’s Modified Eagle Medium F12 (Gibco) and sliced frontally into 200-μm sections with a McIlwain Tissue Chopper (Ted Pella, Inc.). Slices were cultured on PET membranes (353090; Corning) on a steel mesh (FE228710; Goodfellow) in Advanced Dulbecco’s Modified Eagle Medium F12 with 1% penicillin-streptomycin (P4333; Sigma), 15% fetal calf serum, and 0.1 g/L of vitamin C at 37 °C in 5% CO_2_ humidified atmosphere. Explants were treated with aphidicolin (2.0 μg/mL in dimethyl sulfoxide [DMSO]; Santa Cruz Biotechnology); 10μM BrdU was added after 3 or 23 h; and explants were fixed at 5 or 25 h in 4% paraformaldehyde (PFA) for 4 h at room temperature. Explants were photographed under a stereo zoom dissection microscope with brightfield optics at the 3- and 23-h time points (to avoid the trivial shape perturbation sometimes caused by addition of the BrdU-containing medium). For FAK inhibition, explants were treated with 1µM PF-573228 (Cayman Chemicals) in place of aphidicolin.

### Tissue Preparation for Staining

For mT/mG cellular/nuclear morphometric analyses or actin/myosin staining, whole embryos were fixed in 4% PFA for 4 h at room temperature. After a phosphate-buffered saline (PBS) wash, heads were embedded in 0.49 g of gelatin (type B bovine; Sigma-Aldrich), 30 g of albumin, 20 g of sucrose, and 3.5 mL of glutaraldehyde in 100 mL of PBS. Gelatin blocks were refixed in 4% PFA at 4 °C overnight and frontally sliced on a vibratome (VT1000S; Leica) at 90-µm thickness.

### Immunofluorescence and Imaging

For BrdU staining, antigen retrieval (DNA denaturation) was performed with 10mM sodium citrate (pH6) at 95 °C for 20 min, permeabilized in 0.1% Triton X-100 (T8787; Sigma) in PBS (PBST) for 3 × 10 min, then blocked with 20% goat serum (G6767; Sigma) in PBST for 30 to 60 min at room temperature. For P-cadherin staining only, 20% donkey serum (D9663; Sigma) was used instead of goat. Specimens were incubated with primary antibodies at 4 °C overnight. Primary antibodies were as follows: anti-RFP (1:500, 600-401-379; Rockland Immunochemical), goat anti-P-cadherin (1:200, AF761; R&D Systems), rabbit anti-non-muscle myosin IIB (1:200, 909901; BioLegend), and rat anti-BrdU (1:200, ab6326; Abcam). After six 1- to 2-h PBST washes, specimens were incubated at 4 °C overnight with Alexa Fluor–conjugated secondaries (Life Technology). Nuclei and F-actin were counterstained with DAPI (4′,6-diamidino-2-phenylindole; 1:5000, 62247; Thermo Fisher Scientific) and Alexa Fluor 488/635 Phalloidin (1:500, A12379, A34054; Invitrogen), respectively. Specimens were washed 6 times with PBST (1 to 2 h per wash) and then mounted on glass slides with 50% glycerol (356352; Calbiochem) in PBS. Z-stacks were acquired by a confocal microscope (TCS SP5; Leica) with oil immersion 40× and 63× objectives.

### Morphometric Analyses

Slice explants were imaged on a dissecting microscope with transmitted light brightfield optics. The contour of the basal side of the epithelium was manually traced in Fiji/ImageJ (Schindelin et al. 2012) and landmarks marked on the contours (Appendix Fig. 1). Landmark coordinates were entered into the geometric morphometrics package MorphoJ (Klingenberg 2011) for Procrustes scaling (to separate size differences from shape differences) and statistically tested with the multiple permutation test (1,000 permutations).

### Cellular/Nuclear Analyses

Cellular/nuclear measurements used 40× z-stack images (0.21-μm steps) of tooth germs from E13.5 to E15.5 mT/mG mice. Only basal cells not undergoing mitosis (but including other cells with minimal basal contact) were measured. In E13.5, the corners in the bottom one-third of the tooth bud were considered cervical loops. Cells were measured in the optical slice containing their maximum cross-sectional area to avoid grazing artifacts. Morphologic features were measured with Fiji tools. Nuclear position was defined as follows: (distance between centroid and cell base midpoint) / cell height. Basal width was defined as the width of attachment to the basal membrane, and apical width was measured perpendicularly to the cell axis at a site 20% below the cell apex (Appendix Fig. 2). Sample numbers are in the Appendix Table.

### Cell Division Orientation Analysis

For cell division orientation, z-stack confocal images from mT/mG mice stained with DAPI were analyzed. Only anaphase and telophase stages were selected. The acute angle relative to the basal lamina (90° = spindle perpendicular to the basal lamina) was measured in Fiji.

### Statistics

All statistical analyses, except for cell division axis, were conducted with SPSS 24.0 (IBM). Comparison of tissue dimensions in the proliferation inhibition experiment was by Student’s *t* test. Comparison of multiple embryonic stages in cellular/nuclear measurements was analyzed by analysis of variance only (no post hoc tests). For cell division orientation, Mardia-Watson-Wheeler tests (angle counterpart to the Mann-Whitney *U* test, sensitive to the mean and variance differences, although somewhat more to mean differences) were performed with R 3.5.0 (R Core Team 2013) and the package “circular” (Agostinelli and Lund 2011). *P* < 0.05 was considered statistically significant.

## Results

### Proliferation Inhibition Does Not Prevent Bud-to-Cap Morphogenesis

**Figure 2.**
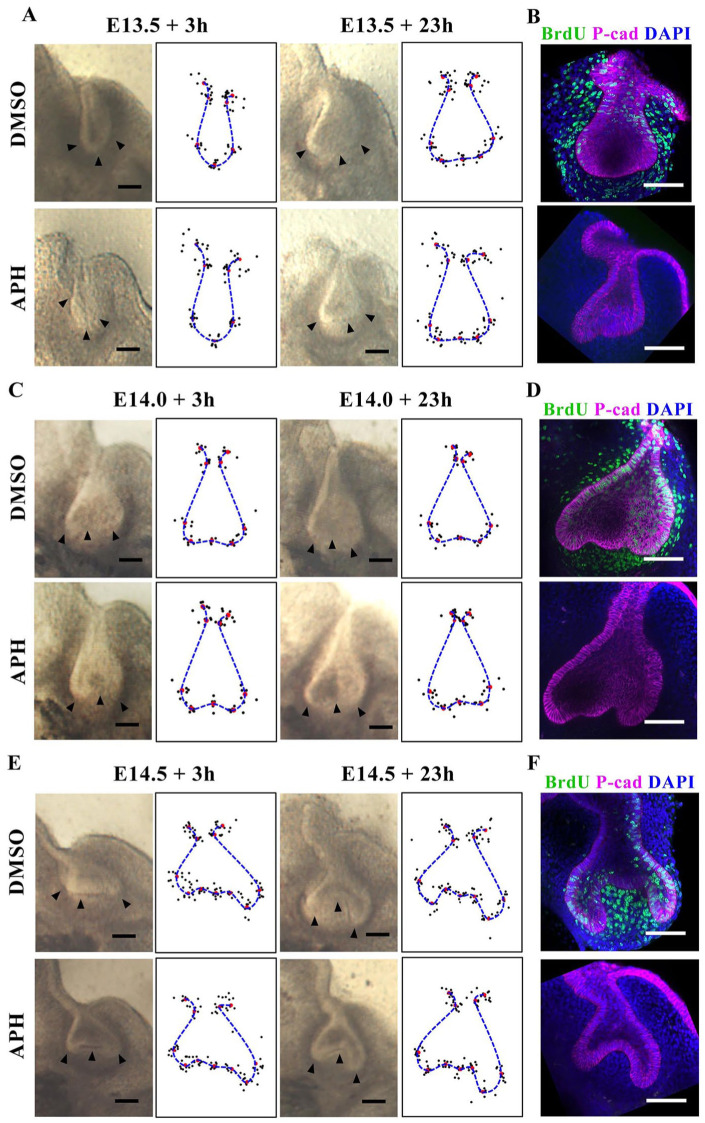
Proliferation inhibition does not inhibit tooth explant bud-to-cap morphogenesis. (**A**, **C**, **E**) Typical brightfield images of tooth explants with or without aphidicolin (APH) at the times indicated with corresponding epithelial landmark and contour plots for multiple samples (*n* = 10 explants from 3 litters for E13.5 and E14.5, *n* = 6 explants from 1 litter for E14.0). Small black dots show positions of individual landmarks; red dots show average position of each landmark; and blue dashed line indicates average contour. (**B**, **D**, **F**) Confocal images of explants shown in panels A, C, and E were labeled for a further 2 h with BrdU (green), confirming that proliferation was inhibited by APH. Buccal is left in all panels. Scale bar = 100 μm.

To directly test the proposition that tooth bud-to-cap morphogenesis is caused by differential proliferation alone, we applied a proliferation inhibitor, aphidicolin, to mouse molar tooth explants. Such explants faithfully recapitulate normal morphogenesis in culture (Alfaqeeh and Tucker 2013; Panousopoulou and Green 2016). Preliminary experiments showed that cultures with aphidicolin were healthy for 1 d but deteriorated after 2 d of incubation (not shown). We therefore aphidicolin-treated explants and controls from E13.5, E14.0, and E14.5 embryos and imaged them after 3 and 23 h to test for effects on morphogenesis, followed by a further 2-h incubation with BrdU label to control for inhibition of proliferation. We confirmed inhibition of proliferation at the early time point, before significant morphogenesis had taken place (Fig. 1B), as well as at the final time point. We found that at all stages tested, the treated explants were substantially smaller than controls, showing growth arrest consistent with complete proliferation arrest (Appendix Fig. 3). Unexpectedly, despite the size difference, the epithelial shape changes were remarkably normal: nonproliferating tooth germs underwent the transition from smoothly rounded buds at E13.5 to more or less triangular shapes by E13.5 + 23 h (Fig. 2A, B) and from the triangular shapes at E14.5 to a clear cap shape by E14.5 + 23 h (Fig. 2E, F). Shape change from E14.0 to E14.0 + 23 h was less obvious (Fig. 2C, D). Qualitatively, the changes were very similar to those of the controls without inhibitor. To test this quantitatively, we used well-established geometric morphometrics (MorphoJ; Klingenberg 2011) to compare the epithelial contours. We found no statistically significant difference (*P* < 0.05, multiple permutation *T*^2^ test) between control and proliferation-inhibited shape. This strongly suggests that the models for bud-to-cap morphogenesis that depend exclusively on differential proliferation do not reflect events in vivo. This finding raised the following question: if not proliferation, what does drive the bud-to-cap transition?

### Cell Shape, Nuclear Position, and Spindle Orientation Analyses Reveal Basal Expansion in Cervical Loops and Basal Contraction in IDE

**Figure 3.**
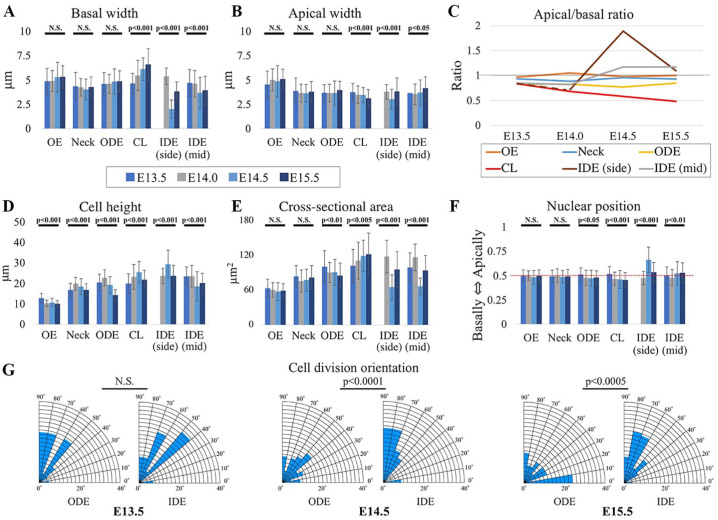
The cellular/nuclear shape analysis of epithelial cells of tooth germs shows basal expansion in the cervical loops (CLs) and basal contraction in the inner dental epithelium (IDE) during bud-to-cap transition. Measurements are from middle optical sections of membrane-labeled cells. (**A–C**) CL cells show a significant increase in basal width and a slight decrease in apical width (columnar to wedge-shape change) from E13.5 to E15.5, while IDE (side) cells were significantly contracted basally at E14.5. (**D**, **E**) Cell heights and areas show mostly increases but an area decrease in IDE at E14.5. Values are presented as mean ± SD. (**F**) Apicobasal position of nuclei in basal cells shows central position except at IDE (side), where nuclei were more apical at E14.5. (**G**) Spindle to lamina angles were all vertical to oblique (i.e., 45° to 90°) at E13.5 but were more randomly orientated in the outer dental epithelium (ODE) at E14.5 and E15.5. *n* = 60 cells/region/stage for panels A–F, *n* = 38–87 in panel G. See Appendix Table for sample number details.

Most epithelial bending mechanisms involve cell shape changes from columnar to wedge shaped (Pearl et al. 2017). To find out whether such cell shape changes occur in the tooth bud-to-cap transition, we quantified apical and basal dimensions, cell heights, and nuclear positions in the different regions of the epithelium at the critical developmental stages. The results are shown in Figure 3. Noting that some cells may shift among defined regions during development, we found that during this period, the apical:basal size ratio in the cervical loops (red line in Fig. 3C) declined steadily, concomitant with deepening invagination of the cervical loops. The ratio decreased partly due to apical reduction (Fig. 3B) but more to basal expansion (Fig. 3A). The most dramatic change in the apical:basal ratio, however, was in the IDE on either side of the enamel knot, which we call the “side IDE” at E14.5 (brown line in Fig. 3C). This corresponded to evagination of the side IDE relative to the bottom of the tooth bud to create the domed lining of the cap. The dramatic IDE ratio change was due to a sharp reduction of the basal size, which outweighed a slight concomitant apical decrease. This reduction was so extreme that the bases of many of these cells were at the resolution of conventional confocal microscopy, so the values plotted for their basal width and apical:basal ratio are maximum and minimum limits, respectively. IDE cells’ mitotic spindles seemed to be more vertically restricted than outer dental epithelium spindles at E14.5 and E15.5 (Fig. 3G) perhaps because the cells also become highly columnarized, although regulated orientation (to generate apical daughter cells, adding overlying stellate reticulum) could also be involved. Together the measurements shown in Figure 3 reveal apical contraction and basal expansion in the cervical loops and basal contraction in the IDE during the bud-to-cap transition.

### Actin/Myosin Localization Indicates Basal Constriction in IDE

**Figure 4.**
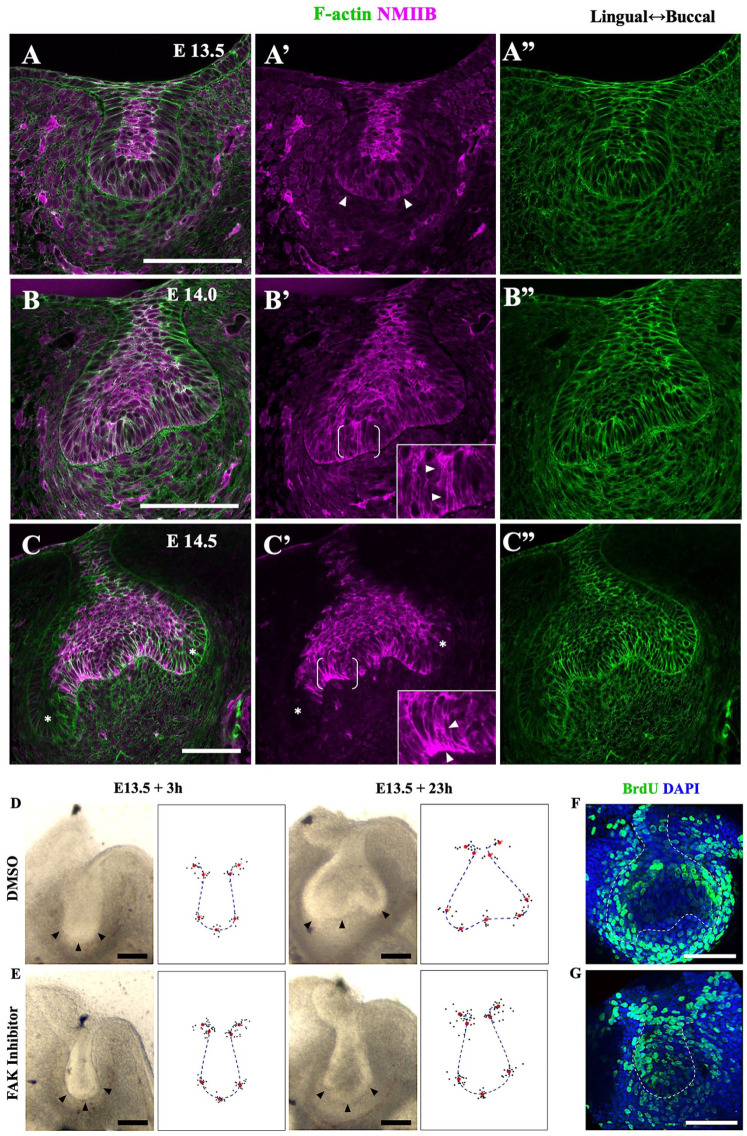
Confocal images of myosin IIB and F-actin in tooth germs show markers of basal relaxation in the cervical loops and basal constriction in the inner dental epithelium during bud-to-cap transition. Confocal images of fixed mandible slices stained for F-actin (green) and nonmuscle myosin IIB (NMIIB; magenta) at E13.5 (A–A′′), E14.0 (B–B′′), and E14.5 (C–C′′). At E13.5, NMIIB is accumulated throughout the suprabasal cells of the bud and sometimes weakly on either side of the base of the bud (arrowheads in A′). (B) At E14.0, NMIIB localizes in the suprabasal cells as well as the basal surface of the flattened bottoms of tooth germ and more highly throughout some inner dental epithelium cells (arrowheads in inset close-up of bracketed region). Strong coaccumulation of NMIIB and F-actin is sparsely found at apical sites of basal cells, particularly around prospective cervical loops and the circumferential regions of enamel knot. (C) At E14.5, there is an obvious enrichment of NMIIB and F-actin at the basal sides of inner dental epithelium, which corresponds to the peripheral enamel knot where basally narrowing cells are aligned (arrowheads in inset close-up of bracketed region). NMIIB is depleted in the outer dental epithelium and cervical loops (asterisks) (**D, E**) Typical brightfield images of tooth explants with or without focal adhesion kinase (FAK) inhibitor at the times indicated with corresponding epithelial landmark and contour plots for multiple samples (*n* = 16 and 8 for inhibitor treated and controls, respectively, from 3 litters. Small black dots show positions of individual landmarks; red dots show average position of each landmark; and blue dashed line indicates average contour. (**F, G**) Typical confocal images of explants labeled for a further 2 h with BrdU (green) show that proliferation persists in FAK inhibitor–treated explants. Buccal is left in panels D–G. Scale bars = 100 μm.

To determine whether the observed cell shape changes were driven by active actomyosin mechanisms, we used fluorescent phalloidin to detect filamentous actin and immunofluorescence against myosin light chain, followed by confocal imaging, noting that mechanical tension is well correlated with total myosin levels in other systems (Streichan et al. 2018; Guy Blanchard, University of Cambridge, personal communication). At E13.5, myosin and actin were both relatively enriched in the suprabasal cells of the bud (Fig. 4A–A′′; consistent with its actively intercalating to make and then narrow the bud neck; Panousopoulou and Green 2016). Occasionally we saw slightly elevated myosin in regions at the base of prospective IDE cells at the bottom of the bud (Fig. 4A′, arrowheads), although this did not show up in averaged quantitations, since it was apparent in only a few sections per specimen (Appendix Fig. 4A, B). At E14.0, there was a slight enrichment of myosin throughout the flattening bottom of the tooth germ (the prospective IDE and enamel knot), smooth basally and punctate, and co-localized with actin apically (white foci in Fig. 4B). Some cells in the side IDE showed even higher actin and myosin throughout (Fig. 4B′, inset arrowheads). The outer dental epithelium was relatively depleted for myosin at this stage. Finally, at E14.5, there was myosin enrichment in the basally narrowing cells of the IDE, particularly basally, and concomitant depletion in the cervical loop and outer dental epithelium (Fig. 4C–C′′, Appendix Fig. 4). Taken together, these results show 1) basal actin and myosin enrichment coincident with basal contraction, indicating basal constriction in the IDE cells, and 2) slight basal depletion and apical elevation of myosin in the outer cervical loops, suggesting some basal relaxation and apical constriction there.

### Sensitivity of Bud-to-Cap Transition to FAK Inhibition Links It to Other Evaginations with Basal Constriction Mechanisms

A well-studied example of basal contraction is the formation of the midbrain-hindbrain boundary constriction (Gutzman et al. 2008), which requires cell matrix adhesion mediated by FAK (Gutzman et al. 2008; Gutzman et al. 2018). To test whether the bud-to-cap transition involves similar mechanisms, we applied the FAK inhibitor PF-573228 to bud-stage explants. We found that, unlike aphidicolin, this clearly arrested epithelial morphogenesis: when controls had progressed to the cap-stage morphology, the FAK inhibitor–treated explants remained bud shaped (Fig. 4D–G). The treated explants were somewhat smaller, although not as small as the aphidicolin-treated explants (Appendix Fig. 3B vs. 3A), but they did incorporate BrdU (Fig. 4G), unlike the aphidicolin-treated explants, indicating at least some cell proliferation. Together these results showed that even if proliferation and/or cell size may have been somewhat reduced by the FAK inhibitor, there was a clear effect on the bud-to-cap morphogenesis. This finding suggests a common mechanism with basal constriction and other contexts. As a side observation, we found that by applying an inhibitor of Rho kinase, an activity that is involved very broadly in cell shape regulation, buds in explants became very enlarged and grossly deformed, rendering interpretation difficult.

## Discussion

In this work, we demonstrated in molar tooth slice explants that apparently normal epithelial shape change from bud to early cap is resistant to cell proliferation inhibition. This gross morphogenesis is therefore unlikely to be driven exclusively (and possibly at all) by differential cell proliferation, which has been proposed in previous models. Although there are caveats with explant experiments and the use of inhibitors, the persistence of morphogenesis during well-controlled inhibition of proliferation is hard to explain away as an artifact of explantation or off-target inhibitor effects. Meanwhile, the proliferation inhibition was well controlled: initiated before any shape change, persisting to the end of the experiment, and resulting in complete growth arrest. Although a previously published proliferation-driven model (Marin-Riera et al. 2018) matched a detailed cell-tracking data set (Morita et al. 2016; Marin-Riera et al. 2018), the results reported here establish that the proliferation is likely to be secondary to, rather than the cause of, the observed morphogenesis during the specific stages that we tested.

We investigated alternative mechanisms that are based on cell shape change. Such changes were invisible to previous computational models because they treated the tissue as a continuous material or as being made of cells in the form of undeformable particles. Myosin was enriched where the cell domains got smaller, and it was depleted where they expanded, indicating active cell-autonomous mechanisms, especially during basal contraction in IDE adjacent to the enamel knot. In *Drosophila*, tension is quantitatively associated with total myosin (Streichan et al. 2018), and we consider this to be a good indicator of active constriction. Myosin phosphorylation is sometimes associated with activation, but we found anti-phosphomyosin immunofluorescence somewhat unreliable. Importantly, we showed that inhibition of FAK is sufficient to arrest bud-to-cap morphogenesis, linking the latter to basal constriction–dependent evagination at the midbrain-hindbrain border (Gutzman et al. 2018) and suggesting that further investigation of signals shown to be active in that system might also be acting in the tooth.

Can our results be reconciled with proliferation-based models and experiments? Mechanosensing can regulate proliferation, so the latter could easily be downstream of the cell-autonomous shape changes that we describe. Proliferation would thus facilitate normal morphogenesis even if not required for it. Additionally, while our proliferation-inhibited explants showed largely normal morphogenesis, subtle changes in the cell sizes and lineage distributions may have compensated for the lack of differential proliferation. Furthermore, we analyzed a narrow stage range, and it is possible that differential proliferation becomes increasingly important in later cap, bell, and cusp formation.

**Figure 5.**
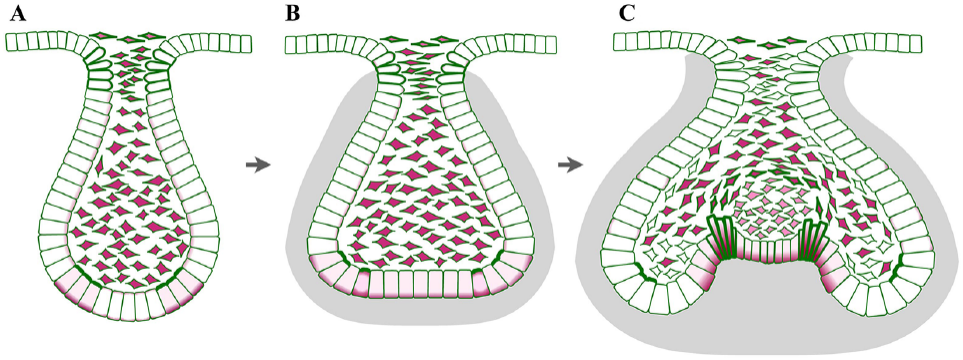
Schematic summarizing cell shape and cytoskeletal changes during bud-to-cap transition. (**A**) Bud-stage molar has elevated actin (green) in the elongated cells of the neck (associated with prior cell intercalation) and high myosin (maroon) in all suprabasal cells. Localized elevation of basal myosin, moderately in most of the cell (pink) and high at the basal end (maroon), in cells at the bottom of the bud on either side of the midline prefigures basal constriction of some cells. (**B**) Cells at the base of the bud have elevated myosin as this prospective inner dental epithelium flattens. The mesenchyme (gray) begins to condense around the epithelium. (**C**) Actin and myosin are sharply elevated in cells of the inner dental epithelium, which radically change their shape, becoming highly columnar and narrowing basally through basal constriction to evaginate the epithelium on either side of the central enamel knot. This creates the cap shape while surrounding mesenchyme constrains the cervical loops to point downward.

We focused on epithelially autonomous mechanisms, but experiments in which removal of the condensing mesenchyme caused the cervical loops to splay outward at mid- to late cap stages have shown clearly that physical constraint by the surrounding mesenchymal condensation is important for correct morphogenesis. This is perfectly compatible with our interpretations, since those experiments were conducted at slightly later stages and mesenchyme removal reoriented the cervical loops but did not relax them or the epithelial bends adjacent to the enamel knot (Morita et al. 2016; Marin-Riera et al. 2018). A combination of autonomous proliferation-independent epithelial bending by basal constriction within an enclosing mesenchymal capsule thus provides a satisfying explanatory physical dual mechanism for molar morphogenesis from bud to bell stage (Fig. 5).

The tooth germ is an outstanding model for developmental organogenesis and has played a major role in understanding gene action and epithelial-mesenchymal signaling. Its morphogenesis is no less interesting, and our identification of basal relaxation and constriction in the cervical loops and IDE, respectively, is a first step toward integrating genetic and signaling aspects with the physical cell behaviors that make this organ.

## Author Contributions

S. Yamada, contributed to design, data acquisition, analysis, and interpretation, drafted and critically revised the manuscript; R. Lav, contributed to design, data acquisition, analysis, and interpretation, critically revised the manuscript; J. Li, contributed to conception, design, data acquisition, critically revised the manuscript; A.S. Tucker, contributed to design and data interpretation, critically revised the manuscript; J.B.A. Green, contributed to conception, design, data analysis and interpretation, drafted and critically revised the manuscript. All authors gave final approval and agree to be accountable for all aspects of the work.

## Supplemental Material

DS_10.1177_0022034519869307 – Supplemental material for Molar Bud-to-Cap Transition Is Proliferation Independent Supplemental material, DS_10.1177_0022034519869307 for Molar Bud-to-Cap Transition Is Proliferation Independent by S. Yamada, R. Lav, J. Li, A.S. Tucker and J.B.A. Green in Journal of Dental Research
