# Supplementary material for: Molar Bud-to-Cap Transition Is Proliferation Independent
Source: J Dent Res. 2019 Aug 8;98(11):1253–61. doi: 10.1177/0022034519869307 (PMC6761786; doi:10.1177/0022034519869307)
Supplement: DS_10.1177_0022034519869307 – Supplemental material for Molar Bud-to-Cap Transition Is Proliferation Independent [file DS_10.1177_0022034519869307.pdf]

Molar Bud-to-Cap Transition Is Proliferation Independent

S. Yamada, R. Lav, J. Li, A.S. Tucker, and J.B.A. Green

Appendix

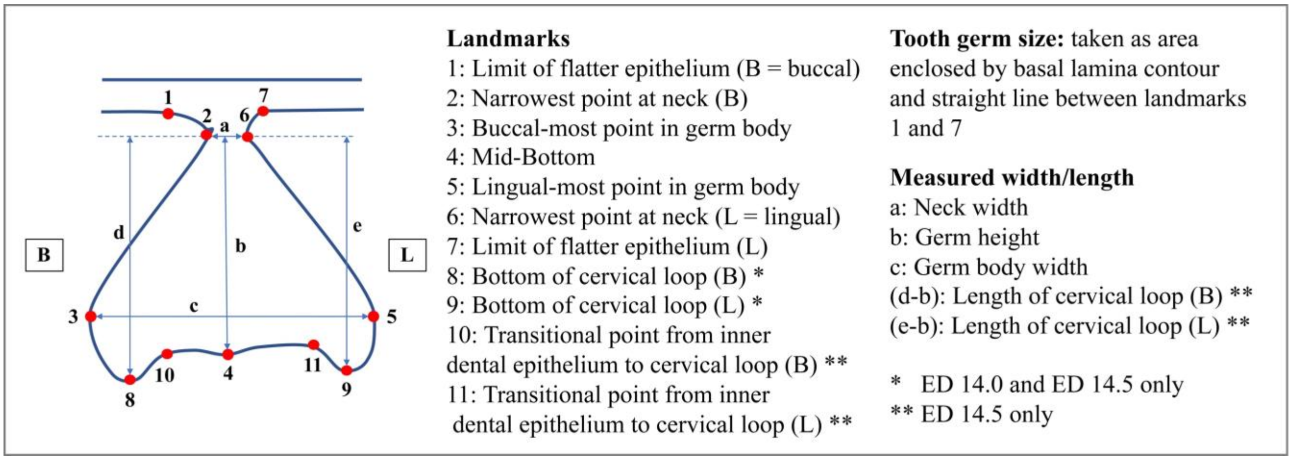

Appendix Fig. 1. Landmarks and dimensional measures used for quantifications in Appendix Fig. 3.

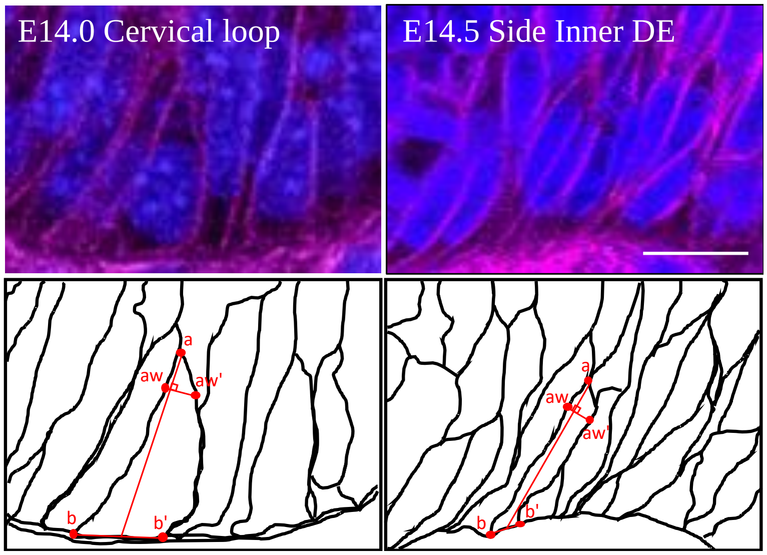

Appendix Fig. 2. Cell Shape measurements**.** Upper panels show example confocal sections of molar regions indicated from mTmG embryos with membranes labelled by membrane-Tomato fluorescent protein (magenta) and nuclei stained with DAPI (blue). Lower panels show cell outlines with example measurements of basal attachment (b-b'), cell apex (a) and apical width (aw-aw') at 20% below the apex along the cell axis (line from apex to midpoint of base). Note that every cell to be measured was inspected at multiple z-sections to resolve any ambiguities (e.g. checking that only cells that made contact with the basal lamina were considered) and measured at its largest section to avoid grazing artefacts.

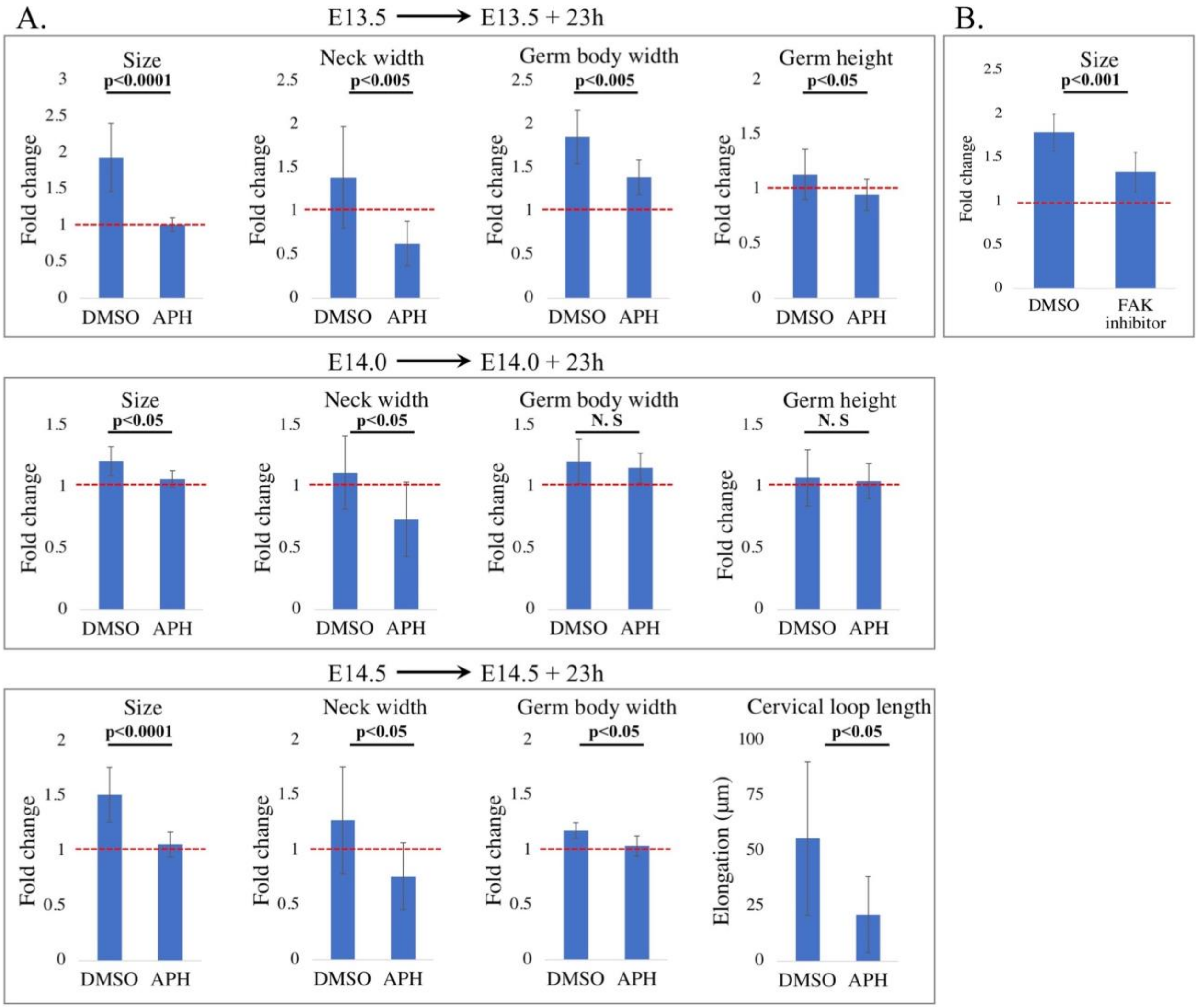

Appendix Fig. 3. The quantification of morphological changes during explant culture with and without aphidicolin treatment. Fold-change in the dimensions indicated, based on the landmarks in Fig. S3, before and after explant culture with or without aphidicolin. The explants cultured with APH possessed smaller tooth germs in size (area enclosed by the epithelial contour and a straight line between landmarks 1 and 7), neck width, germ body width and height at all stages as well as cervical loop length at E14.5 +23h. Error bars = s.e.m.

A.

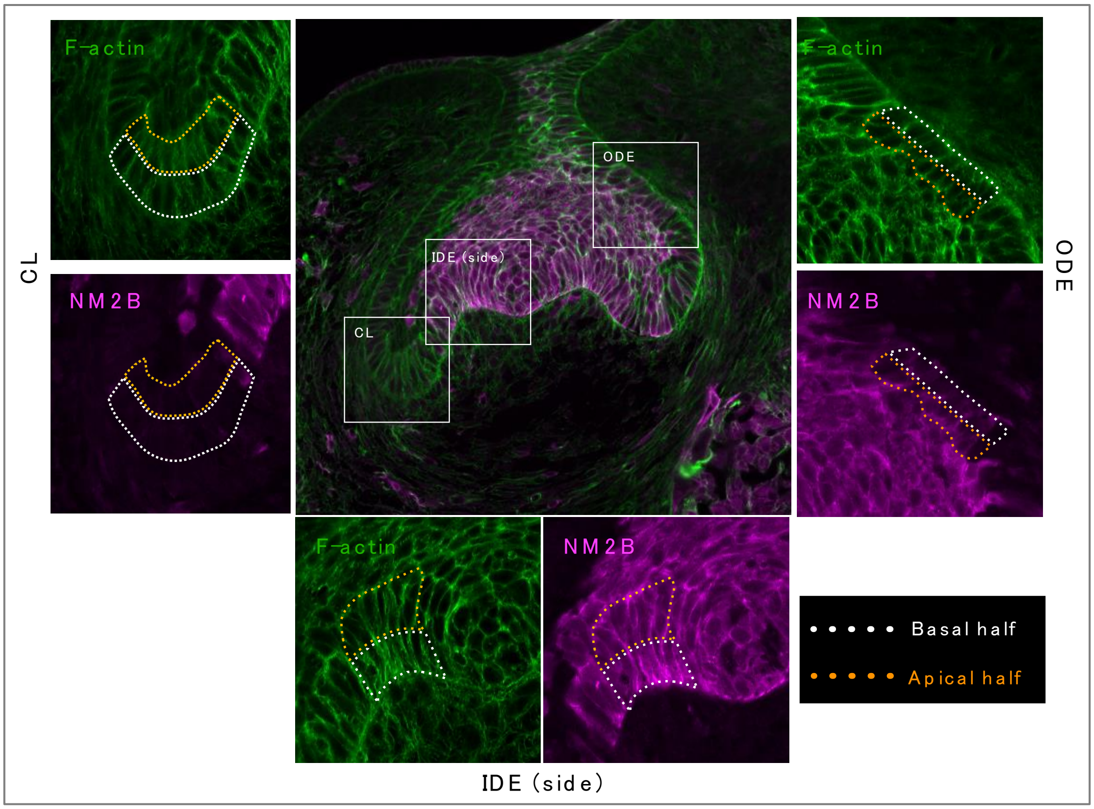

B.

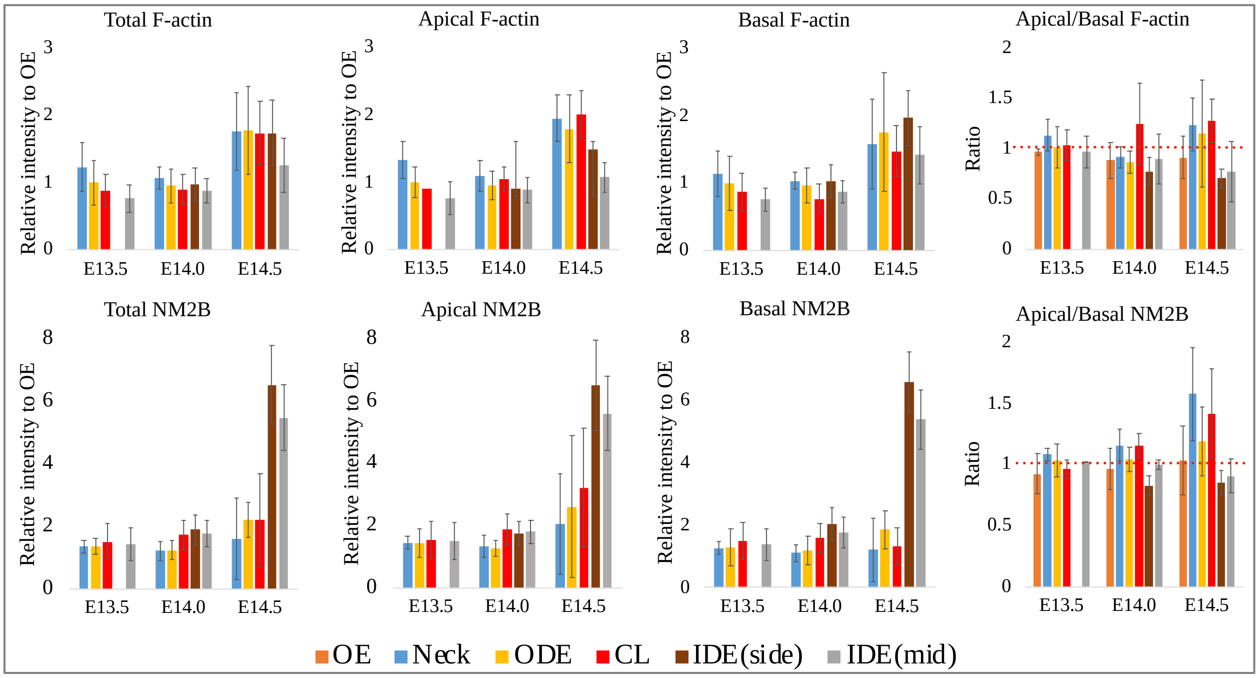

Appendix Fig. 4. Quantification of fluorescence signals from apical and basal regions of basal epithelial cells in the tooth germ during the bud-to-cap transition. A. Fluorescence intensity

was measured in Fiji using manually drawn regions of interest in the regions as shown. B. Histograms show the ratio of the average pixel intensity of each region to that of OE (measured lingually and buccally and averaged for each image). Each bar represents the mean of six measurements (lingual and buccal regions from 3 different specimens from 2 or 3 different litters). Error bars = s.d.

Appendix Table. Sample numbers for cell shape/nuclear position/spindle orientation analysis

	Stage	Number of tooth germs	Number of basal cells OE	Neck	ODE	CL	IDE side	IDE mid	n (/stage)	Total n
Cellular/nuclear shape analyses	E13.5	6	60	60	60	60	-	60	300	1380
	E14.0	6	60	60	60	60	60	60	360	
	E14.5	6	60	60	60	60	60	60	360	
	E15.5	6	60	60	60	60	60	60	360	
	Stage		ODE				IDE		n (/stage)	Total n
Spindle orientation	E13.5	10	20				15		35	210
	E14.5	11	53				35		88	
	E15.5	8	34				53		87	
